# Electric field-enhanced backscatter interferometry detection for capillary electrophoresis

**DOI:** 10.1038/s41598-024-52621-3

**Published:** 2024-01-24

**Authors:** Miyuru De Silva, Robert C. Dunn

**Affiliations:** Department of Chemistry, University of Kansas, Lawrence, KS 66047 USA

**Keywords:** Bioanalytical chemistry, Sensors

## Abstract

Backscatter interferometry (BSI) is a refractive index (RI) detection method that is easily integrated with capillary electrophoresis (CE) and is capable of detecting species ranging from inorganic ions to proteins without additional labels or contrast agents. The BSI signal changes linearly with the square of the separation voltage which has been used to quantify sample injection, but has not been explored as a potential signal enhancement mechanism in CE. Here we develop a mathematical model that predicts a signal enhancement at high field strengths, where the BSI signal is dominated by the voltage dependent mechanism. This is confirmed in both simulation and experiment, which show that the analyte peak area grows linearly with separation voltage at high field strengths. This effect can be exploited by adjusting the background electrolyte (BGE) to increase the conductivity difference between the BGE and analyte zones, which is shown to improve BSI performance. We also show that this approach has utility in small bore capillaries where larger separation fields can be applied before excess Joule heating degrades the separation. Unlike other optical detection methods that generally degrade as the optical pathlength is reduced, the BSI signal-to-noise can improve in small bore capillaries as the larger separation fields enhance the signal.

## Introduction

Capillary electrophoresis (CE) is a versatile separation technique that has become increasingly popular due to its simplicity, low cost, and high performance^1,2^. In general, CE separates analytes based on their charge-to-size ratio and is capable of analyzing a wide range of analytes. A large body of work has shown that analytes from inorganic ions^3^, amino acids^4^, carbohydrates^5^, lipids^6^, and nucleic acids^7^, to large macromolecular complexes like polymers and proteins^8^ are compatible with CE analysis. Techniques have been developed, moreover, to not only quantify analyte levels, but also measure binding constants^9–11^, rate constants^12,13^, and other fundamental parameters^14,15^. This breadth of capabilities has made CE a flexible and cost-effective tool for many analytical applications.

Recently, advances in lab-on-a-chip technology has further led to integration of electrophoresis with microfluidics to develop miniaturized devices capable of separating and detecting analytes in small channels^16–18^. Here the choice of detection method is critical to maintain adequate performance as dimensions are reduced.

A plethora of techniques have been adapted for detection in CE including UV-Vis^19^, fluorescence^20^, Raman^21^, and electrochemical methods^22^, to name a few. To detect analytes that are not optically or electrochemically active, species can often be derivatized for detection. This, however, can increase the workflow, cost, and length of analysis while often degrading performance due to label stability or coverage. Alternatively, many indirect detection methods have been developed for analytes lacking easily exploited detection mechanisms. These approaches use contrast agents added to the BGE to visualize depleted analyte zones. These approaches, however, generally result in a reduction in sensitivity and can lead to irregularities in the baseline^23^.

Universal detectors exploit attributes inherent to all analytes and are capable of detecting species in their native states without derivatization steps. Mass spectrometry (MS), for example, has been coupled successfully with CE to directly detect analytes^24^. This is a very powerful approach, but can be limited by the expense and complexity of the instrumentation; and introduces significant hurdles for future miniaturization. Other universal detectors like conductivity and refractive index, on the other hand, are easily miniaturized, cost effective, and easily implemented.

Conductivity detectors sense the difference in conductivity between the BGE and analyte zones and are thus universal detectors^25^. The introduction of capacitively coupled contactless conductivity detection (C4D) has simplified the geometry by enabling measurements from outside the separation channel^26^. Excitation and pickup electrodes are positioned near the capillary wall to deliver and receive an AC signal. The attenuated AC signal received at the pickup provides information about the conductivity between the electrodes. C4D detection has found widespread applications in CE and is particularly useful for amino acid analysis^27,28^. Optimizing the electrode geometry and separation however, is critical for minimizing capacitive coupling, which can degrade performance by increasing background levels^29^.

Refractive index (RI) is another universal detector that is generally thought to sense the difference between the refractive index of the analyte band and the BGE. RI detection in electrophoresis dates back to the very first electrophoresis experiment performed by Tiselius in 1937^1^. With the introduction of modern CE, various strategies have been introduced over the years to integrate universal RI detection with CE. These methods include offline detection using techniques like surface plasmon resonance^30,31^, photonic ring resonators^32^, and whispering gallery mode resonators^33^. Online detection methods include the use of photonic crystals^34^, retro-reflected beam interference^35,36^**,** and integrated ring resonators^37^. Among the many online RI detection methods, backscatter interferometry (BSI) has proven to be a sensitive and robust approach, capable of detecting analytes with submicromolar detection limits^38,39^.

In BSI, a laser is focused into the detection zone of a CE capillary where the curvature of the capillary creates a fan-like interference pattern around the detection point. Part of the back scattered fringe pattern is collected and projected onto a suitable detector^40–42^. Changes in the RI of the solution within the detection volume alters the optical pathlength and shifts the position of the interference fringes, which is detected as the BSI signal. This approach has been shown capable of detecting refractive index changes on the order of 10^–7^ RIU^41^ and has been implemented in the detection of inorganic ions^41,43–45^, amino acids^38^, proteins^39,46^, binding interactions^47–51^, and system zones to study their effects on analyte peaks^52^. The detection optics are inexpensive and easily miniaturized, as recently demonstrated in a study using an optical pickup head manufactured for DVD players as a BSI detector in CE^44^.

RI detection, however, is generally considered a less sensitive method for analyte detection and new approaches using compensation^53–55^ and wavelength modulation^43^ have been introduced to improve detection performance. One promising route that has not been explored, to our knowledge, involves the separation voltage^56^. Studies have shown that the BSI signal is very sensitive to the applied field, increasing with the square of the separation voltage. This has been attributed to the effects from Joule heating^57^ and/or the Kerr effect^45^. Since both mechanisms predict a linear increase in BSI signal with the square of the separation voltage, teasing out the contributions from each is complicated^45^. While previous work has shown the utility of this effect for quantifying sample injections in CE, its impact on BSI signal strength and possible signal enhancement has yet to be fully explored^45^.

In this study, we develop a model of the BSI signal that includes both the refractive index and the effects of the applied separation voltage. We use this model to simulate electropherograms and compare them with experimentally measured electropherograms to validate the approach and develop strategies for improving BSI detection. We test the possibility that at large field strengths, the BSI signal is dominated by the electric field component which may provide a promising path for enhancing detection performance. We also examine the possibility of improving detection through the judicious selection of background electrolyte (BGE), using conditions that optimize the conductivity difference between the BGE and analyte zones. Finally, we compare BSI electropherograms as the separation channel is reduced from 50 to 25 µm, which enables larger separation voltages before the deleterious effects of Joule heating. This study introduces electric-field-enhanced BSI for sensitive, universal detection in microfluidic electrophoretic devices.

## Results

**Figure 1:**
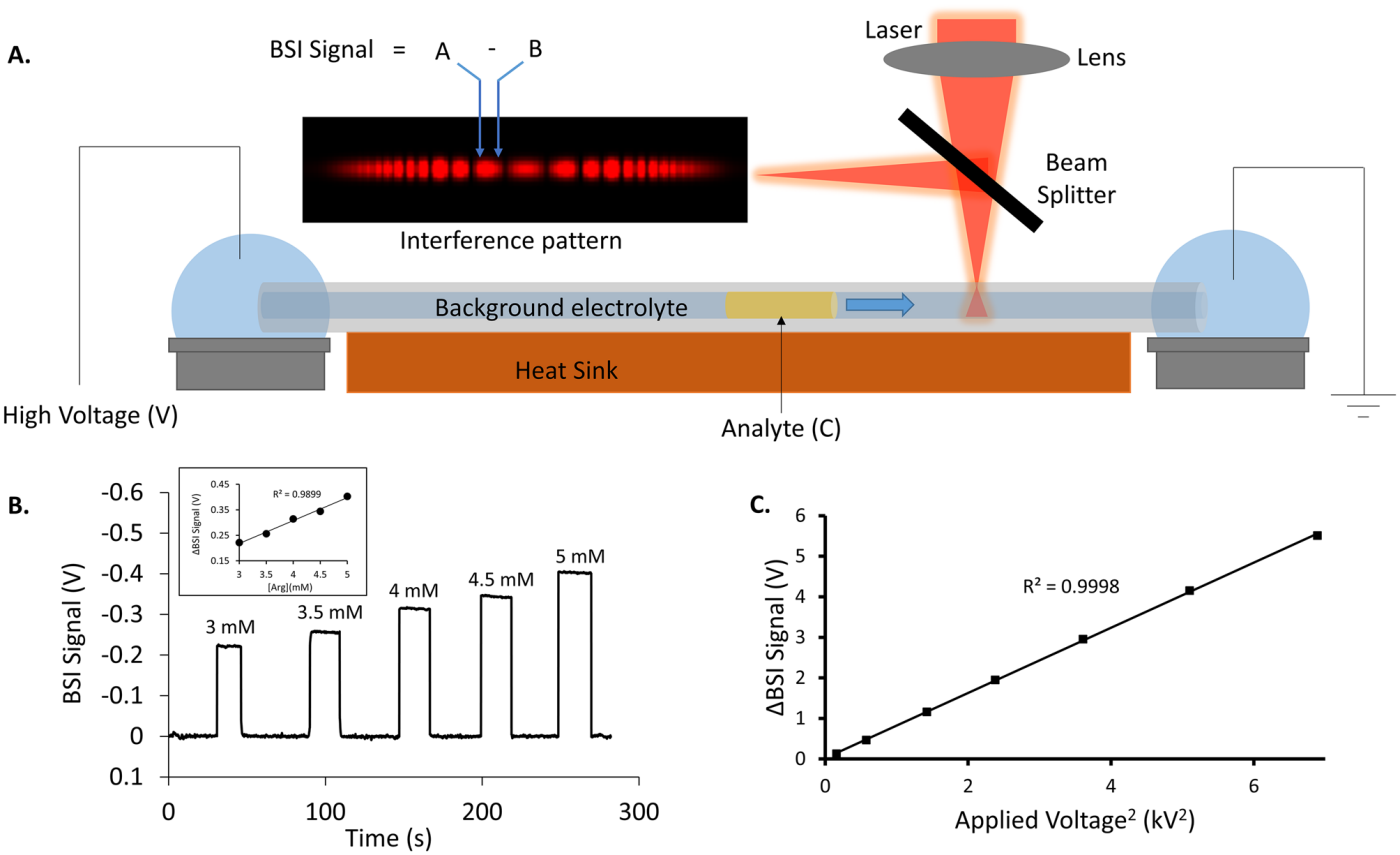
(**A**) Schematic diagram of the planar CE platform using BSI detection. The separation capillary is mounted on a temperature controlled heat sink and covered along its length with a thermal paste (not shown), except at small regions at the capillary ends and at the detection window. Laser excitation is focused into the detection zone of the capillary and the resulting interference pattern is directed towards a split photodiode detector. The two quadrants of the photodiode are positioned in the interference pattern as shown, with the differential output producing the BSI signal. (**B**) Plot of the BSI signal as the concentration of arginine in a solution flowing past the detector is increased as indicated. Inset shows the linear response of the ΔBSI signal with concentration. (**C**) Linear relation between the increase in BSI signal and square of the applied voltage. The BSI signal increases with field strength and the error bars are within than the data points shown.

The BSI detection scheme depicted in Fig. 1A is similar to that previously reported by our group^38^. Laser light delivered through a single-mode optical fiber is focused into the detection zone of the capillary using a 20x objective lens. A beam splitter above the capillary directs the backscattered interference pattern towards a split photodiode detector, with the two active areas positioned in the resulting fringe pattern as shown in Fig. 1A. Any change in the refractive index of the solution filling the detection volume alters the optical pathlength and shifts the interference fringes^44^. These shifts are detected in the differentially amplified output of the split photodiode, which produces the recorded BSI signal.

Assuming external factors are held sufficiently constant during an electrophoretic separation, two main mechanisms contribute to the change in the refractive index (Δn) measured by the BSI signal. These contributions arise from the composition of the solution in the detection volume (Δn_C_) and the separation field itself (Δn_E_), with the total BSI signal change given by:

1 $$\Delta n={\Delta n}_{C}+{\Delta n}_{E}.$$

To illustrate each contribution, Fig. 1B shows the BSI response as the concentration of arginine (Arg) flowing past the detector is increased, thus changing the solution refractive index (Δn_C_). Figure 1C shows the effect of the applied field on the BSI signal (Δn_E_). As shown previously, there is a linear relationship between the BSI signal and the square of the applied separation voltage^45^. Without specifying the mechanism of this effect (thermal and/or Kerr effect), this effect can be expressed as:

2 $${\Delta n}_{E}={K}^{\prime}\cdot {E}_{x}^{2},$$

where K’ is a constant and *E*_*x*_ is the local electric field strength in the detection volume. *E*_*x*_ can be rewritten in terms of the local conductivity using:

3 $${E}_{x}=\frac{J\left(x,r,\theta \right)}{\kappa \left(x,r,\theta \right)}\approx \frac{J}{{\kappa }_{x}},$$

where J is the current density in the separation channel, *κ*_*x*_ is the local conductivity, x denotes the position along the capillary length and r and θ indicate the radial position relative to the central axis of the separation channel. In the presence of excess Joule heating, a large temperature gradient can develop between the central axis and capillary wall, leading to radial variations in J (x, r, θ) and κ(x, r, θ). In the limit of minimal Joule heating where the capillary is symmetrically thermostatted, we can assume the temperature gradient is small^58^ and E_x_ simplifies to that shown in Eq. (3). For the present study, all separations were carried out in a BGE producing low currents <20 µA, which generates less than 1 W/m of power. Moreover, as previously reported the capillary is affixed to a heat sink and covered along its length with a thermal conducting paste (except for small regions at the capillary ends and detection window). This combined with the thin capillary wall (15 µm) leads to efficient heat dissipation from the central channel. Based on the power delivered and capillary geometry, we estimate a temperature variation of less than 0.13 °C between the central axis and capillary wall, justifying the approximation shown in Eq. (3)^59^.

Substituting (3) into (2) leads to:

4 $${\Delta n}_{E}={K}^{\prime}\cdot {J}^{2}\cdot {\left(\frac{1}{{\kappa }_{x}}\right)}^{2},$$

which shows that the current density (local conductivity) can contribute to changes in the BSI signal. Combining Eqs. (1) and (4) leads to the total BSI signal change:

5 $$\Delta n={\Delta n}_{C}+{K}^{\prime}\cdot {J}^{2}\cdot {\left(\frac{1}{{\kappa }_{x}}\right)}^{2}.$$

This can be re-written in terms of the total field strength using:

6 $$\Delta n={\Delta n}_{C}+{K}^{\prime}\cdot {E}_{t}^{2}\cdot {{\upkappa }_{t}}^{2}\cdot {\left(\frac{1}{{\kappa }_{x}}\right)}^{2},$$

where *E*_*t*_ is the total field applied between the anode and cathode and *κ*_*t*_ is the conductivity of the solution filling the capillary.

Equation (6) is useful for understanding how the BSI signal responds to analyte bands passing through the detection volume during a CE separation. In the absence of diffusion, we can approximate the analyte band as a square pulse. The amplitude of the pulse reflects the difference in BSI signal from the analyte zone and BGE given by:

7 $$\Delta n=\left[{n}_{BGE}+{K}_{BGE}^{\prime}\cdot {E}_{t}^{2}\cdot {\left(\frac{{\kappa }_{t}}{{\kappa }_{BGE}}\right)}^{2}\right]-\left[{n}_{Analyte}+{K}_{Analyte}^{\prime}\cdot {E}_{t}^{2}\cdot {\left(\frac{{\kappa }_{t}}{{\kappa }_{Analyte}}\right)}^{2}\right] .$$

While the constant of proportionality used in Eq. (2) depends on the composition of the material, at the modest concentrations used in CE and similarities between BGE and analyte zones, we can use the approximation ${K}_{Analyte}^{\prime}\approx {K}_{BGE}^{\prime}={K}^{\prime}$ to simplify Eq. (7) to:

8 $$peak height=\left({n}_{BGE}-{n}_{Analyte}\right)+ {K}^{\prime}\cdot {\kappa }_{t}^{2}\cdot \left[{\left(\frac{1}{{\kappa }_{BGE}}\right)}^{2}-{\left(\frac{1}{{\kappa }_{Analyte}}\right)}^{2}\right]\cdot {E}_{t}^{2},$$

where now we have recognized that the total change in measured refractive index gives us the analyte peak height in the BSI signal. Equation (8) suggests that the BSI signal can be enhanced by the field strength through the second term, which responds to the difference in conductivity between the BGE and the analyte zone. It is also worth noting that both Δn_C_ (first term) and Δn_E_ (second term) can be positive or negative depending on the magnitude of their individual parameters (*n*_*BGE*_*, n*_*Analyte*_*, κ*_*BGE*_*, and κ*_*analyte*_).

While this model is intuitive, the absence of diffusion makes peak height a problematic parameter to compare with the results of experiment where diffusion takes place. Peak area, on the other hand, is less sensitive to the effects of diffusion and can reasonably be used to compared with the results of experiments.

Multiplying Eq. (8) by *W/µE*_*t*_, where *W* is the full width of the analyte zone, *µ* is its mobility, and *E*_*t*_ is the total field strength, leads to the following equation for peak area:

9 $$peak area=\frac{W}{\mu \cdot {E}_{t}}\left({n}_{BGE}-{n}_{Analyte}\right)+\frac{W}{\mu }\cdot {K}^{\prime}\cdot {\kappa }_{t}^{2}\cdot \left[{\left(\frac{1}{{\kappa }_{BGE}}\right)}^{2}-{\left(\frac{1}{{\kappa }_{Analyte}}\right)}^{2}\right]\cdot {E}_{t} .$$

Finally, we can rewrite Eq. (9) in a simplified form using the following definitions:

10a $$\alpha =\left({n}_{BGE}-{n}_{Analyte}\right),$$

10b $$\beta ={K}^{\mathrm{^{\prime}}}\cdot {\kappa }_{t}^{2}\cdot \left[{\left(\frac{1}{{\kappa }_{BGE}}\right)}^{2}-{\left(\frac{1}{{\kappa }_{Analyte}}\right)}^{2}\right],$$

10c $$\gamma =\frac{W}{\mu },$$

Which leads to:

11 $$peak\,area = \left( {\gamma \cdot \alpha \cdot \frac{1}{{E_{t} }}} \right) + \left( {\gamma \cdot \beta \cdot E_{t} } \right).$$

The first term in Eq. (11), (γα/E_t_), is common to any CE detection method and reflects the decrease in peak area with faster migration times^60^. As field strength increases, analytes pass through the detection zone faster leading to smaller peak areas. The second term in Eq. (11), (γβE_t_), is specific to refractive index detection in CE and causes the peak area to change linearly with the applied electric field.

**Figure 2:**
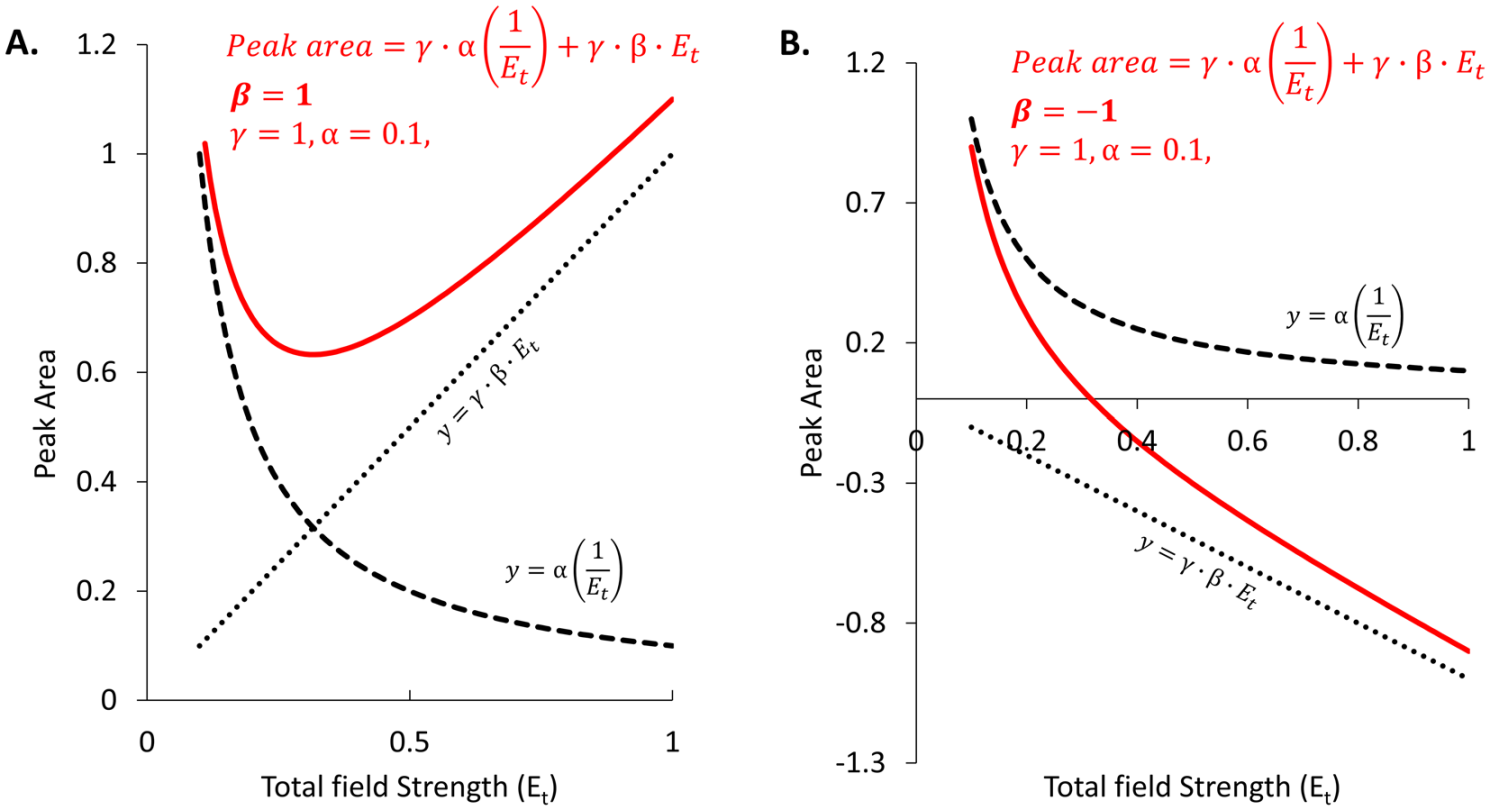
Peak area versus separation field strength calculated using the equations in red for conditions where the β term is positive (β = 1) (**A**) and negative (β = -1) (**B**). Other parameters were set to: α = 0.1 and γ = 1 in both plots. (**A**) The dashed and dotted lines show the expected change in peak area with separation field strength for the first and second terms of the equation shown in red, respectively. The solid red line shows the expected trend in peak area when both terms are included. (**B**) Similarly, the dashed and dotted lines show the expected change in peak area with separation field strength for the first and second terms of the equation shown in red, respectively. Again, the solid red line shows the expected behavior with both terms are included. In both cases, the results show that the second term (field enhancement) dominates at higher separation fields and leads to a linear relationship between analyte peak area and applied field.

To explore trends in the BSI signal using Eq. (11), we first assume that α is always positive. Under this assumption, the β term can be either positive or negative relative to α, depending on the conductivities of the BGE and analyte zone. This leads to plots of theoretical peak area versus total field strength such as those shown in Fig. 2. Figure 2A shows plots for an analyte/BGE system with a positive β term while Fig. 2B shows similar plots for a system with a negative β term.

The dashed lines in both Fig. 2A and B plot the first term of Eq. (11), showing the expected decrease in peak area as total field strength is increased and analytes increasingly rush past the detector. The dotted line in each panel plots the second term of Eq. (11) and shows the linear relationship between peak area and field strength. This enhancement term has a positive slope in Fig. 2A reflecting the positive β value and a negative slope in Fig. 2B reflecting the negative β value. The red curve in each panel shows the expected trend in signal when both phenomena are present.

These plots clearly show that in both cases the linear term dominates at higher field strengths and leads to a mechanism for enhancing the overall BSI signal. In addition, for the negative β value case in Fig. 2B (α is positive), the data shows that the BSI peak is expected to transition from a positive going peak at low fields to a negative going peak at higher fields.

**Figure 3:**
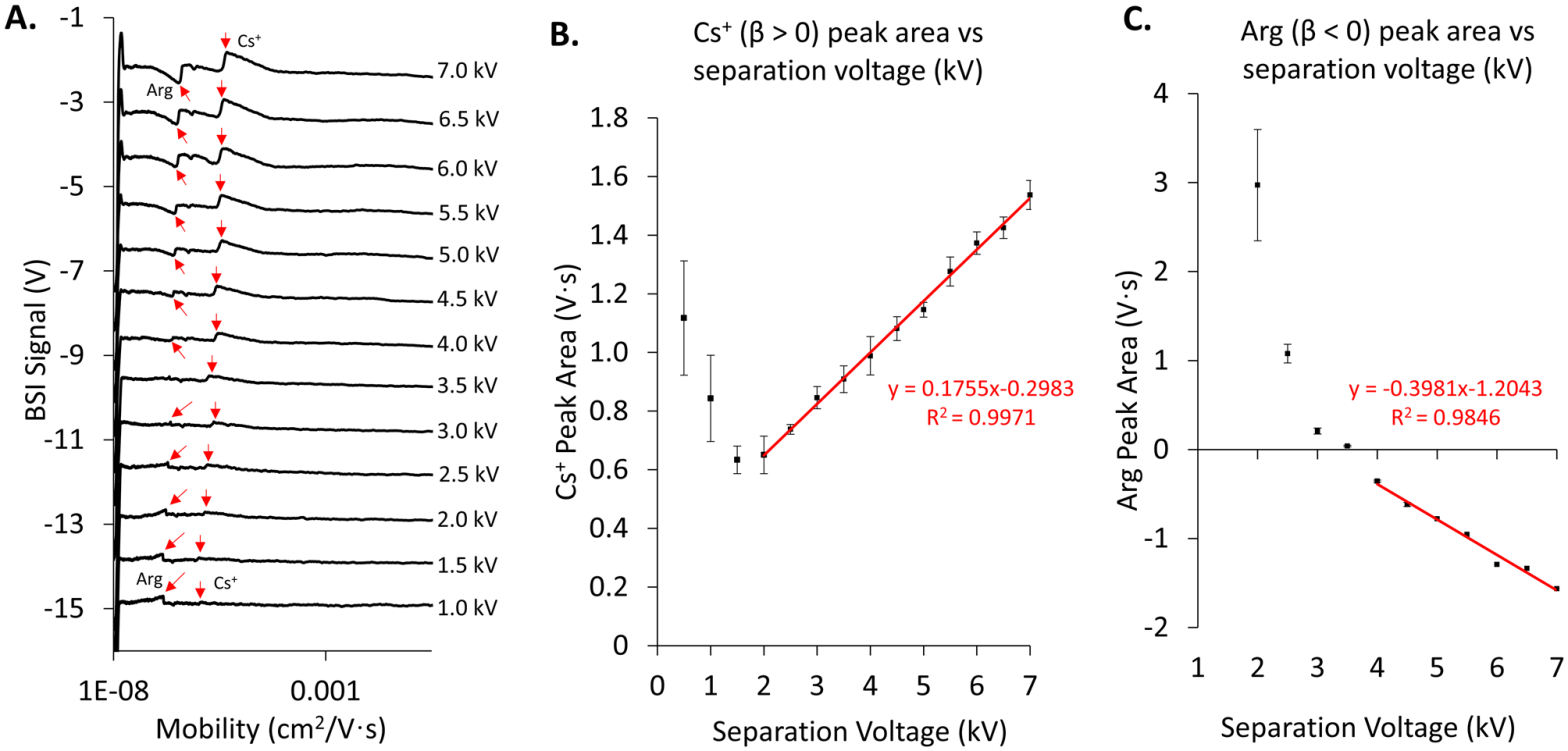
(**A**) Electropherogram regions measured for the separation of Cs + (positive β value) and Arg (negative β value) as a function of separation voltage. Migration times have been converted to mobilities to facilitate comparisons. Arg peak is shown by the first arrow in each electropherogram while Cs peak is indicated by the second arrow. At low voltages, both peaks are positive. As the separation voltage increases, Arg transitions to a negative peak while Cs + stays positive at all separation voltages. Peak area versus separation voltage for Cs + (**B**) and Arg (**C**) extracted from the experimental measurements shown in (**A**). The trends in peak area for a positive (**B**) and negative (**C**) β values agree with the plots presented in Fig. 2.

To experimentally verify the proposed model in Eq. (11), a series of CE separations were carried out on mixtures containing 10 mM Arg and 10 mM CsCl. The samples were hydrodynamically injected into the capillary and separated in a 150 mM glycine buffer at pH 3.43. All experimental parameters were kept constant (injection time, temperature, etc.) between runs, with only the separation field varied. Fig. 3A compares the same region of the electropherograms with the x-axis converted to effective mobilities to facilitate comparisons between the Arg and Cs^+^ peaks.

At low separation fields, Fig. 3A shows that both Arg and Cs^+^ are positive going peaks. In this regime, the first term of Eq. (11) dominates the BSI signal and reflects the difference in refractive index between the BGE and the analyte zone. As the separation field is increased, the peak area for Cs^+^ is seen to increase as the field enhancement term in Eq. (11) begins to take over and dominate the BSI signal. The peak associated with Arg, on the other hand, initially decreases with increasing field strength, passes through zero, and then reappears as a negative going peak at high separation fields. Figure 3B and C plot peak area (integrated by migration time) vs separation voltage for both Cs^+^ and Arg. These curves can be compared with the theoretical curves plotted in Fig. 2 for the overall refractive index signal (red trace). The curve for Cs^+^ in Fig. 3B resembles the curve plotted in Fig. 2A for an analyte with a positive β value, while that for Arg in Fig. 3C resembles the trend in Fig. 2B for an analyte with a negative β value.

**Figure 4:**
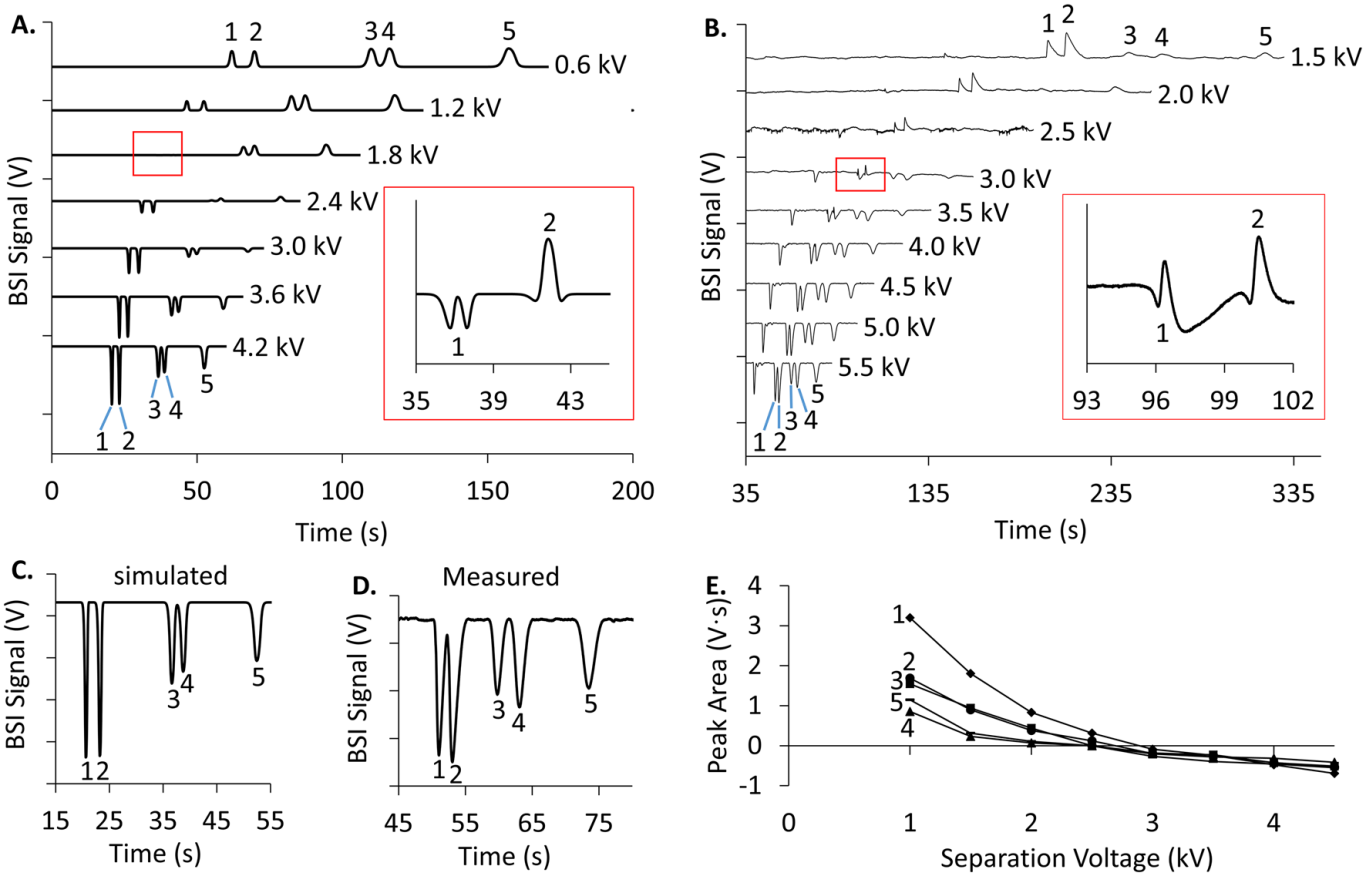
(**A**) COMSOL simulations for the separation of 5 amino acids (1-Lys, 2-Arg, 3-Gly, 4-Ala, 5-Glu) at 1 mM each using 4 M acetic acid (AcOH) at pH 2.1 using a length-to-detector of 8 cm. BSI signals were calculated using Eq. (12) and electropherograms were simulated at the separation voltages indicated representing field strengths ranging from 60 to 420 V/cm. The Inset shows the electropherogram simulated at 180 V/cm which captures the transition of Lys (1) and Arg (2) from positive going to negative going, with the doublet of Lys arising from the positive contribution of refractive index and negative field enhancement. (**B**) Experimental electropherograms measured for the separation of the same 5 amino acids separated in the same 4 M AcOH (pH 2.1) BGE. Electropherograms were measured at the indicated separation voltage representing field strengths ranging from 150 to 550 V/cm. The Inset shows the experimental validation for the Lys peak splitting observed during the transition as seen in the simulation. (**C**) and (**D**) Comparison of the simulated BSI signal strength (420 V/cm) and measured BSI signal strength (550 V/cm), respectively, supporting the validity of Eq. (12). (**E**) Measured peak area versus separation voltage extracted from the experimental electropherograms for the 5 amino acids.

While the proposed model is developed in the absence of diffusion, this restraint can be relaxed to include diffusion by replacing constants in Eq. (6) with functions as follows:

12 $$BSI \; Signal=\frac{\partial n}{\partial C}\cdot C\left(x,t\right)+{K}^{\prime}\cdot {J}^{2}\cdot {\left(\frac{1}{{\kappa }_{x}\left(x,t\right)}\right)}^{2},$$

where *C (x,t)* is the analyte concentration at position *x* and time *t*. Although it is difficult to find an exact solution for *C(x,t)* and *κ*_*x*_*(x,t)*, simulation programs like COMSOL using the zone electrophoresis module can readily simulate these parameters^61^. COMSOL simulations for the electrophoretic separation of 5 amino acids (1 mM each) in 4 M acetic acid (AcOH) at pH 2.1 are compared with experiment in Fig. 4 over a range of separation field strengths. We approximate ∂n/∂C to be constant within this concentration range and use experimentally measured average values for this and K’ to calibrate the response in the COMSOL simulations. The total field strength was adjusted in the simulations using changes in the current density *(J)*.

Figure 4A and B show the simulated and measured electropherograms, respectively, for the separation of 5 amino acids as a function of separation field. The simulated electropherograms in Fig. 4A reveal a transition from positive going peaks at low separation voltages to negative going peaks at high field strengths, with a subsequent increase in signal amplitude. This trend is consistent with the field-induced signal enhancement mechanism and suggests that all the amino acids have a negative β value in this BGE. The inset in Fig. 4A captures the moment that the Lys and Arg peaks transitions from positive going to negative going. The apparent doublet for Lys (peak 1) arises from the positive going refractive index contribution (Eq. 10a) combined with the negative going conductivity component (Eq. 10b). A similar transition can be seen forming in the Arg peak (peak 2).

Figure 4B shows experimentally measured electropherograms for the same 5 amino acids in the same BGE over a range of separation field strengths. Consistent with the simulations, these electropherograms show a progression from positive going peaks at low separation fields to negative going peaks at high fields. There is also a significant increase in signal amplitude at higher fields, consistent with the enhancement mechanism. The inset for the 3 kV separation shows the transition for Lys and Arg as they go from positive-going to negative-going, similar to the behavior seen in the simulations. The experimental measurements also reveal a system peak that migrates before the amino acids.

Figure 4C and D compare the simulated and experimental electropherograms, respectively, indicating that the model can qualitatively predict relative peak heights. Finally, Fig. 4E plots the trends in peak area with separation field for the 5 amino acids. These trends are similar to the model shown in Fig. 2B for analyte/BGE systems with a negative β value.

**Figure 5:**
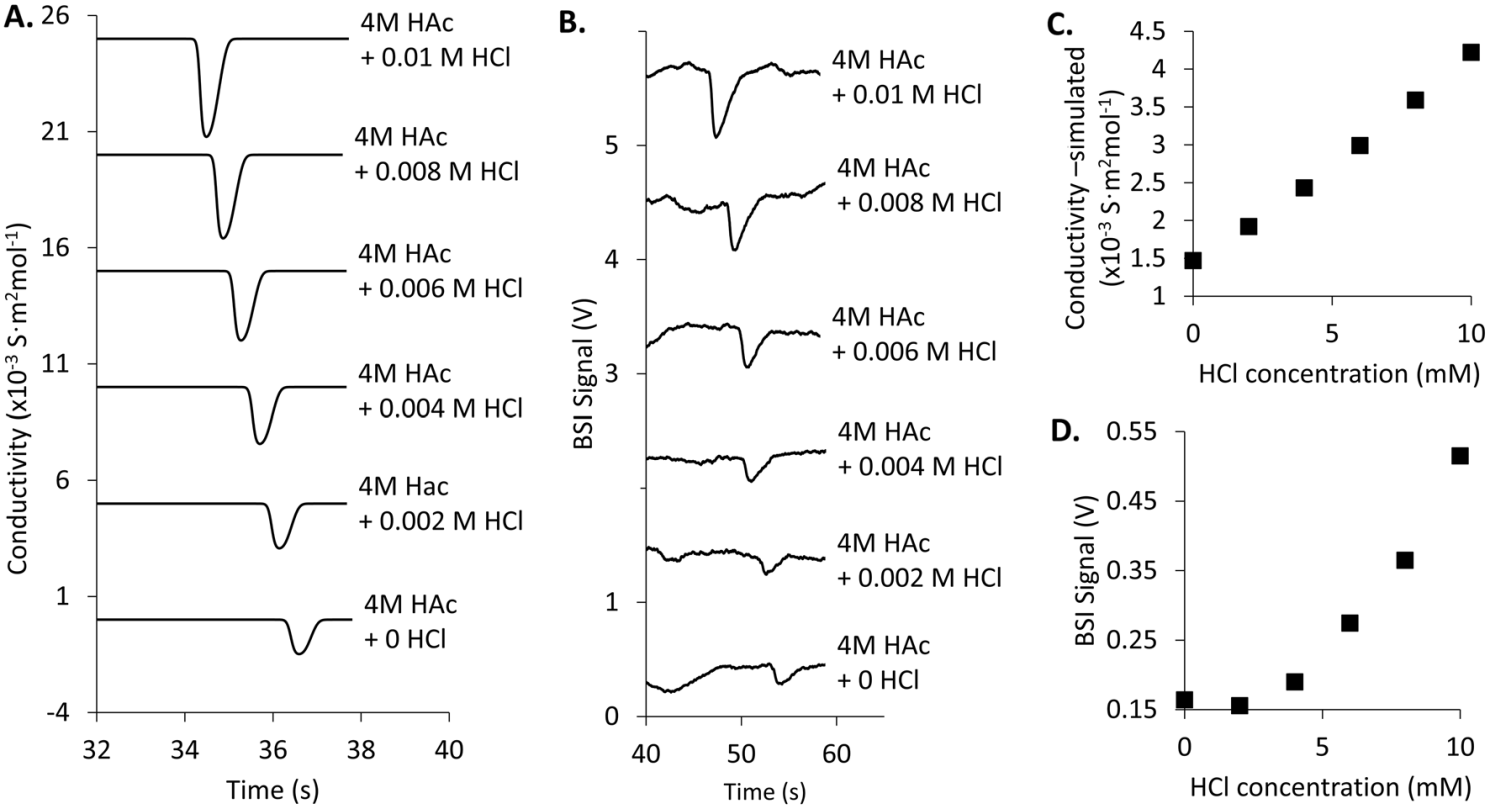
(**A**) Simulated electropherograms (PeakMaster) for 500 μM Arg in a 4 M AcOH (pH 2.1) BGE using a field strength of 400 V/cm and a length-to-detector of 8 cm. The conductivity signal is calculated as HCl is added to the BGE to modify the conductivity of the BGE. Increasing the HCl leads to greater contrast between the BGE and Arg signal, leading to stronger peaks. (**B**) Experimentally measured electropherograms for Arg in the 4 M AcOH (pH 2.1) BGE as a function of HCl level. Using the same 400 V/cm field strength, the BSI signal improves as the conductivity contrast between BGE and analyte zone is increased, consistent with Eq. (8). Plots showing the effects of added HCl on simulated analyte peak conductivity (**C**) and on the experimentally measured peak BSI signal (**D**).

The model summarized in Eq. (6) provides guidance for developing strategies to enhance BSI detection for low concentration analytes. One strategy involves increasing the magnitude of the β value by optimizing the BGE to maximize the conductivity contrast between the analyte zone and the BGE. To test the effectiveness of this approach, electropherograms of 500 μM Arg were measured using a BGE of 4 M AcOH (pH 2.1) into which small amounts of HCl were added to modify the conductivity. Figure 5A shows a series of simulated electropherograms (Peakmaster) that confirm the conductivity signal for Arg grows as the amount of HCl added to the BGE increases^62,63^. Fig. 5B shows experimentally measured electropherograms for the same system using BSI detection. A significant increase in the BSI signal-to-noise is observed as HCl is added into the BGE. The simulated and measured trends in analyte peak height are compared in Fig. 5C and D, respectively. The non-linearity seen in Fig. 5D is difficult to ascribe to a single mechanism given the complexity of the mixture, but certainly has contributions from the squared conductivity term in Eq. (6). This type of BSI enhancement, which relies on conductivity contrast and hence field strength differences between the BGE and analyte zones, is expected to lead to some peak shape asymmetry as observed in C4D detection.

Another strategy for enhancing the BSI signal is by increasing the field strength as shown in Eq. (6). In general, increasing the field strength in CE is desirable to reduce analysis time, but has practical limitations due to excess Joule heating.

In the planar CE approach shown in Fig. 1, the capillary is mounted on a temperature-controlled copper baseplate and its length is covered with a thermal conducting paste (except at the detection zone and capillary ends) to help thermostat the capillary. This combined with the thin wall of the capillary (15 µm) facilitates heat dissipation and mitigates the effects of Joule heating, as discussed elsewhere^38,45^.

Joule heating can also be reduced by decreasing the diameter of the separation channel. While the reduced pathlength of the smaller diameter channel typically degrades optical detection, with BSI detection it can actually improve the signal-to-noise since the high separation fields enhance the BSI signal through the squared-field dependence.

**Figure 6:**
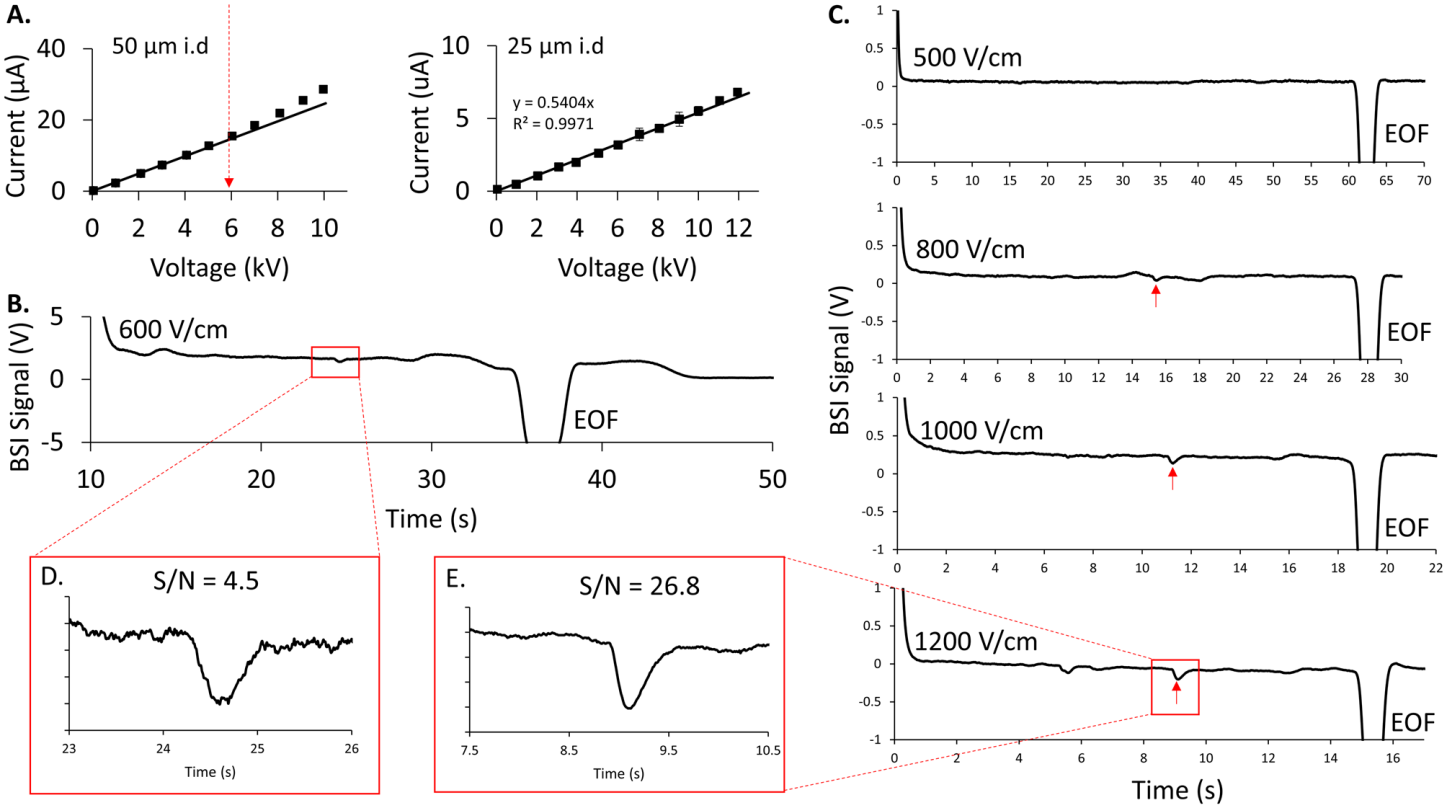
(**A**) Comparison of Ohm’s plots measured in the 50 μm i.d and 25 μm i.d capillaries using a BGE of 20 mM citrate (pH 2.44). The dashed arrow in the 50 μm capillary plot indicates the onset of non-linear behavior reflecting the visible effects of Joule heating. For the 25 μm capillary, the plot remains linear up to the highest applied field strength of 1100 V/cm. (**B**) Experimentally measured electropherogram of 5 μM Arg in 20 mM Citrate (pH 2.44) using a 50 μm i.d capillary at the highest field strength (600 V/cm) measured before the visible effects of Joule heating. (**C**) Series of experimentally measured electropherograms of 5 μM Arg in 20 mM Citrate (pH 2.44) using a 25 μm i.d capillary as the field strength is increased from 500 to 1200 V/cm. The Arg peak amplitude grows (red arrow) as the field strength increases. The Arg peak measured in the large-bore capillary at 600 V/cm (**D**) is compared with the same peak measured in the small-bore capillary at 1200 V/cm (**E**). Even though the optical pathlength decreases, the larger field strength possible in the small-bore capillary leads to large BSI field enhancements and better signal-to-noise as indicated. All measurements used a 8 cm length-to-detector and noise levels were calculated over a 1 s range for both measurements.

To illustrate the field enhancement, electropherograms measured in a 50 μm i.d capillary (80 μm o.d.) are compared with similar measurements in a 25 μm i.d capillary (75 μm o.d.). Figure 6A compares current vs voltage plots for both capillaries when filled with a 20 mM citrate buffer at pH 2.44. The effect of Joule heating is observed in the 50 μm id capillary at 600 V/cm while Joule heating shows negligible effect on the Ohm’s plot in the 25 μm i.d capillary up to the highest measured value of 1100 V/cm.

Figure 6B shows an electropherogram for 5 μM Arg separated in 20 mM citrate BGE (pH 2.44) using the 50 μm i.d capillary. The electropherogram was measured at the highest field strength (600 V/cm) before the effects of Joule heating were observed in the Ohm’s plot. The boxed Area expands the region where the Arg peak is observed.

This is compared with the series of electropherograms shown in Fig. 6C, measured using the 25 μm i.d capillary. With the same sample and BGE, electropherograms are measured as the separation field strength increases from 500 to 1200 V/cm. The arrows denote the location of the Arg peak, which grows as the field strength increases. Fig. 6D and E compare the analyte peak measured in both capillaries, which shows a 6x increase in signal-to-noise for the smaller inner diameter capillary measured at the higher field strength.

These results provide promising routes for optimizing BSI detection in CE. While the use of small diameter channels can be a challenge due to the risk of blockage, these results show significant improvement in BSI detection at the higher field strengths possible. This approach is also particularly promising for applications requiring short separation lengths, where high field strengths and large signal enhancements can be achieved using modest separation voltages.

It should be noted that the BSI signal enhancement scheme outlined here is completely general and capable of improving detection in other systems where a conductivity difference exists between the BGE and analyte bands. As such, it benefits from the extensive studies done previously in developing and optimizing buffers and conditions for C4D detection. Our results show that the field enhanced signal will always dominate at higher field strengths, providing a path towards optimizing detection. The deleterious effects of Joule heating are always a concern as separation fields increase, thus low conductivity buffers are beneficial to fully exploit this mechanism.

## Conclusions

In this study, we explore the influence of the separation field on the BSI signal during CE separations. Based on experimental observations, a mathematical model is developed to describe the BSI signal for a given analyte/BGE system. The model includes contributions from the analyte and BGE refractive index and a term that describes the influence of separation voltage on the BSI signal. At low field strengths, the model shows that the BSI signal is dominated by the differences in refractive index between the BGE and analyte zone. As the separation field strength increases, the field-enhanced term dominates the BSI signal and peak area becomes linearly proportional to the applied separation field. This contribution can be positive or negative depending on the relative conductivities of the BGE and the analyte zone and the model is validated by comparing computer simulations with experimentally measured electropherograms. The model predicts a field-enhancement in the BSI signal that increases with applied separation voltage and conductivity contrast between the BGE and analyte zones. These are confirmed experimentally. Interestingly, we show that BSI detection can actually improve as the separation channel diameter is reduced, due to the larger separation fields and hence enhancements that can be utilized. These results provide guidance for optimizing universal RI detection for microscale, electrophoretic separations.

## Methods

### Chemicals

Cesium chloride, (Fisher Scientific, Fair Lawn, New Jersey), lysine, arginine, alanine, glycine, glutamic acid (Sigma Aldrich St. Louis, Missouri) were used without further purification and dissolved in the appropriate background electrolyte. Glycine buffer (pH 3.43) was prepared by dissolving glycine (Sigma Aldrich, St. Louis, Missouri) in ultrapure water and adjusting the pH using 1 M HCl. 4 M acetic acid (pH 2.1) was prepared by diluting glacial acetic acid (Fisher Scientific, Fair Lawn, New Jersey) with ultrapure water. 20 mM Citrate (pH 2.44) was prepared by dissolving citric acid (Sigma Aldrich, St. Louis, Missouri) in ultrapure water. All solutions were filtered through a 0.22 µm filter, stored at 4 °C, and allowed to warm to room temperature prior to use. All experiments were carried out at 23 °C.

### CE instrumentation

Details of the home-built planar CE instrument shown schematically in Fig. 1 have been discussed elsewhere^38^. Briefly, an uncoated 10 cm total length fused silica separation capillary (50 μm i.d. x 80 μm o.d. or 25 μm i.d. x 75 μm o.d.; VitroCom, Mountain Lakes, NJ) is mounted on a temperature-controlled metal baseplate. The length of the capillary is covered with a thermal conducting paste except at the detection zone and capillary ends. This combined with the thin wall of the capillary (15 µm) facilitates heat dissipation and mitigates the effects of Joule heating^38,45^. For BSI detection, the 647 nm line of an argon-krypton laser (Coherent, Innova 70, Santa Clara, CA) is focused into the detection zone of the capillary, located 8 cm from the inlet. The backscattered interference pattern is directed towards a split photodiode (EG&G Optoelectronics, Quebec, Canada) using a 45° beamsplitter as shown in Fig. 1. The two quadrants of the photodiode are aligned on the interference fringes as shown in Fig. 1 and the differential output amplified (Stanford research systems, SR560) to generate the BSI signal. The gain was set to 200 and a low pass filter at 3 Hz was used for all measurements. Prior to each measurement, the split photodiode was re-aligned on the same fringe location where the initial differential output showed a BSI signal of 0 V. The BSI signal is sent to a data acquisition interface (National Instruments, USB-6001) and recorded using a program written in LabVIEW. Peak areas are calculated using Origin software (OriginLab Corporation).

Fluids are exchanged at the capillary inlet using a rotating sample holder to position the meniscus above vials in the capillary path. Prior to use, an under pressure applied at the capillary outlet is used to flush the capillary with solutions positioned at the inlet. Capillaries are conditioned by flushing for 5 min. each with 1 M NaOH, ultrapure water, and BGE. All samples were injected hydrodynamically by applying an under pressure of 5kPa at the capillary outlet for 1 s. A Spellman CZE1000R high voltage power supply was used to generate the separation voltage, with the inlet reservoir at positive potential and the outlet reservoir grounded for normal polarity separations. The currents measured at the maximum separation voltage during each separation were 10 µA, 20 µA, 18 µA and 7 µA for glycine, acetic acid, citrate (25 µm i.d) and citrate (50 µm i.d) respectively.

### Simulations

Simulations were performed either using the COMSOL (ver 5.5) zone electrophoresis module or Peakmaster (5.4). Details of the simulations along with the analyte and BGE pK_a_ values and mobilities are summarized in the Supplementary Information.

### Supplementary Information

Supplementary Information.

## Data Availability

The data that support the findings of this study are available from the corresponding author upon reasonable request.
