# Supplementary material for: Electric field-enhanced backscatter interferometry detection for capillary electrophoresis
Source: Sci Rep. 2024 Jan 24;14:2110. doi: 10.1038/s41598-024-52621-3 (PMC10808210; doi:10.1038/s41598-024-52621-3)
Supplement: Supplementary file 1 — Supplementary Information. [file 41598_2024_52621_MOESM1_ESM.docx]

**Supporting information**

**Electric Field-Enhanced Backscatter Interferometry Detection for Capillary Electrophoresis**

Miyuru De Silva and Robert C. Dunn*

Ralph N. Adams Institute for Bioanalytical Chemistry

Department of Chemistry

University of Kansas

2030 Becker Drive

Lawrence, KS 66047

Table of contents

S1. Parameters used for COMSOL simulation

S2. Parameters used for Peakmaster simulation

S1. COMSOL simulations were performed using the values shown in the table for the capillary length, length to detector and the concentrations of each analyte as well as the background electrolyte constituents. The pKa values and mobilities of each amino acid were taken from a previous report^1^.

| **Parameter** | **Value** |
| --- | --- |
| Total capillary length | 10[cm] |
| Length to detector | 8[cm] |
| Temperature | 25[degC] |
| Acetic acid initial concentration | 4000[mM] |
| Acetic acid pKa | 4.76 |
| Acetic acid mobility | 4.24e-8[m^2/V/s]/F_const |
| Lysine initial concentration | 1[mM] |
| Lysine pKa1 | 1.79 |
| Lysine pKa2 | 9.127 |
| Lysine pKa3 | 10.79 |
| Lysine mobility 1 | 5.51e-8[m^2/(V*s)]/F_const |
| Lysine mobility 2 | 2.86e-8[m^2/(V*s)]/F_const |
| Lysine mobility 3 | 2.64e-8[m^2/(V*s)]/F_const |
| Arginine initial concentration | 1[mM] |
| Arginine pKa1 | 1.58 |
| Arginine pKa2 | 8.919 |
| Arginine pKa3 | 12.48 |
| Arginine mobility 1 | 6.1e-8[m^2/(V*s)]/F_const |
| Arginine mobility 2 | 2.55e-8[m^2/(V*s)]/F_const |
| Arginine mobility 3 | 2.69e-8[m^2/(V*s)]/F_const |
| Glycine initial concentration | 1[mM] |
| Glycine pKa1 | 2.4 |
| Glycine pKa2 | 9.6 |
| Glycine mobility 1 | 3.95e-8[m^2/(V*s)]/F_const |
| Glycine mobility 2 | 3.74e-8[m^2/(V*s)]/F_const |
| Alanine initial concentration | 1[mM] |
| Alanine pKa1 | 2.3 |
| Alanine pKa2 | 9.7 |
| Alanine mobility 1 | 3.95e-8[m^2/(V*s)]/F_const |
| Alanine mobility 2 | 3.32e-8[m^2/(V*s)]/F_const |
| Glutamic Acid initial concentration | 1[mM] |
| Glutamic Acid pKa1 | 9.96 |
| Glutamic Acid pKa2 | 4.324 |
| Glutamic Acid pKa3 | 2.14 |
| Glutamic Acid mobility 1 | 2.87e-8[m^2/(V*s)]/F_const |
| Glutamic Acid mobility 2 | 2.7e-8[m^2/(V*s)]/F_const |
| Glutamic Acid mobility 3 | 5.43e-8[m^2/(V*s)]/F_const |
| ∂n/∂C | 2.158 V/mM |
| K’ | 8.14 x 10^-7^V/V^2^ |

S2. Peakmaster simulations were performed using following parameters

| **Parameter** | **Value** |
| --- | --- |
| Capillary length | 10 cm |
| Length to detector | 8 cm |
| Separation Voltage | 4000 V |
| Electroosmotic marker time | 90 s |
| Polarity | positive |
| Analyte (Arg) concentration setting | s |

References

1. Včeláková, K.; Zusková, I.; Kenndler, E.; Gaš, B., Determination of cationic mobilities and pKa values of 22 amino acids by capillary zone electrophoresis. *ELECTROPHORESIS* **2004,** *25* (2), 309-317.
